# Oseltamivir-induced hepatotoxicity: A retrospective analysis of the FDA adverse event reporting system

**DOI:** 10.1371/journal.pone.0314970

**Published:** 2025-02-25

**Authors:** Lurong Yu, Qiumeng Xiang, Limei Liu

**Affiliations:** 1 College of Traditional Chinese Medicine of Chongqing Medical University, Chongqing, China; 2 Pharmacy Department of Chongqing Youyoubaobei Women and Children’s Hospital, Chongqing, China; National Taiwan Ocean University, TAIWAN

## Abstract

Assessing the potential for oseltamivir-induced liver damage is essential to ensure its safe administration. The aim of this study was to examine the association between hepatotoxicity and oseltamivir use and to describe the features of oseltamivir-induced hepatotoxicity. Data were obtained from the Adverse Event Reporting System of the US Food and Drug Administration (FAERS). Disproportionality and proportionality analyses were performed to evaluate the safety profile of oseltamivir-related hepatotoxicity and the occurrence of hepatotoxicity-related adverse events across sex and age groups. The FAERS recorded 20,340,254 adverse event reports between 2004 and 2023, of which 16,960,996 reports were included in the analysis. We identified 14 types of oseltamivir-related adverse events that were hepatotoxic and showed positive signals. The most frequently reported adverse event was abnormal hepatic function (n =  54), and the most severe adverse event was fulminant hepatitis. Compared with that for male individuals, the reporting odds ratio (ROR) was 0.5 for female individuals; and for male individuals, the ROR, compared with that for female individuals, was 4.19. The median time to hepatotoxic adverse events, excluding mixed liver injury, was <  5 days. Oseltamivir can cause liver toxicity, which is influenced by sex and age. Liver function tests and monitoring for signs of liver disease are crucial when using oseltamivir.

## Introduction

Seasonal influenza, a prevalent respiratory disease, annually affects approximately 20–30% of children and 5–10% of adults worldwide [1,2]. Influenza A and B viruses are the primary strains that contribute to the global impact of influenza [1]. Oseltamivir, a selective neuraminidase (a glycoprotein present on the surface of influenza A and B viruses), inhibits influenza-infected cells by preventing viral release [3,4]. Influenza leads to approximately 200,000 hospitalizations and 36,000 fatalities annually in the United States [5]. In 1999, the US Food and Drug Administration (FDA) first approved oseltamivir for influenza treatment and prophylaxis in children and adults aged > 13 years. The age at initiation of influenza treatment was reduced from 13 years to 1 year in 2000, with approval for prophylaxis in this age category being granted in 2005.

During the 2009 influenza pandemic, oseltamivir was temporarily approved for use in infants aged 1 year. In 2012, the age range was lowered to 2 weeks and prophylaxis was approved for patients aged ≥  1 year [5–7]. Oseltamivir is a leading drug for influenza prevention and treatment and is widely used in clinical practice. The active form of oseltamivir decreases the function of natural glycosidases (neuraminidases) in the body, causing a delayed onset of the harmful effects of neuraminidase inhibitors, such as liver damage [8]. Recently, serious adverse events of hepatic systemic disorders following oseltamivir therapy have been observed.

In 2018, a case of oseltamivir-induced liver injury was reported in a child [9]. The child developed abnormal liver function, with aspartate aminotransferase (AST) and alanine aminotransferase (ALT) levels of 69 U/L (reference range: 15–40 U/L) and 80 U/L (reference range: 9–50 U/L), respectively, after two doses of oseltamivir. The patient presented with malaise and slight yellowing of the skin and sclera. After discontinuing oseltamivir for approximately 2 weeks, both ALT and AST levels steadily increased, with the ALT level increasing to 1,931.5 U/L and the total bilirubin (TBIL) level increasing to 53.3 mmol/L (reference range: 6.0–30.0 mmol/L). The patient was treated with hepatoprotective agents, such as glucocorticoids, glycyrrhizin, and reduced glutathione, and was subsequently discharged from the hospital. Two weeks after discharge, the patient’s liver function normalized.

A 2021 case reported acute cholestatic hepatitis following oseltamivir therapy, with significant elevations in liver enzymes and bilirubin [10,11]. On day 4 of taking oseltamivir, the patient experienced a combination of hepatocellular and cholestatic liver injury, including jaundice, nausea, malaise, and pruritus, followed by anorexia, urine darkening, and liver pain. The AST, ALT, alkaline phosphatase (ALP), and TBIL levels increased to 1,280 U/L (reference range: 1–34 U/L), 1,600 U/L (reference range: 25–65 U/L), 330 U/L (reference range: 50–136 U/L), and 3.5 mg/dL (reference range: 0.4–1.2 mg/dL), respectively. The report did not describe the intervention protocol; however, it indicated that 2 weeks after oseltamivir discontinuation, the clinical and laboratory values were within normal range. In this study, we aimed to retrospectively review the US FDA Adverse Event Reporting System (FAERS) for the incidence of oseltamivir-induced hepatotoxicity compared with that induced by other FDA-approved medications and the timing of its occurrence, for the early identification of oseltamivir-induced hepatotoxicity by clinicians.

## Materials and methods

### Data source

The data used in this study were sourced from the FAERS database, which is accessible to the public. The FAERS data file contains seven datasets: patient demographics and management (DEMO), records of adverse events (REAC), drug details (DRUG), patient outcomes reporting sources, medication use indications, and reporting of medication entry and end dates [12]. We downloaded data from the FAERS database from the first quarter of 2004 to the third quarter of 2023. SAS 9.4 software was used to clean and analyze all downloaded data. This study examined anonymized and publicly accessible human data; therefore, this study did not require patient consent and was reviewed by the Ethics Committee of Chongqing Youyoubaobei Women’s and Children’s Hospital on December 12, 2023.

### Study design and standardization

The DEMO file was processed according to the FDA’s recommended method for duplicate reporting, using CASEID (FAERS case identification number) as the primary criteria, followed by FDA_DT (date the FDA received the case), and finally PRIMARYID (a unique identifier for FAERS reports) for selection and sorting. To ensure accuracy, we selected the most recent FDA_DT when matched against the CASEID [12]. If CASEID and FDA_DT were similar, reports with higher PRIMARYID scores were selected [12]. In the FAERS, negative events are categorized using preferential terms (PTs) from the International Dictionary of Medical Terminology (MedDRA). We used MedDRA (version 26.1) to standardize the preferred terms and access the most recent PT and the system organ classification details from the REAC files. Within the FAERS database, the DRUGNAME field specifies the drug’s name, whereas the PROD_AI field specifies the product’s composition. Oseltamivir was approved by the FDA on October 27, 1999. We screened data using the DRUGNAME and PROD_AI fields with the search commands INDEX(PROD_AI, “OSELTAMIVIR”) OR INDEX(DRUGNAME, “OSELTAMIVIR”) OR INDEX(DRUGNAME, “TAMIFLU”). Filtering was performed based on the liver system organ code (10019805) and conditions that met the criteria for reporting disproportionate signals.

### Statistical analysis

The risk of liver toxicity due to oseltamivir was compared with that of all other drugs during the same period, and the ROR was calculated using disproportionate signal analysis. The ROR represents the level of correlation between exposure to a drug and the likelihood of a specific outcome occurring [13]. A signal of reporting disproportionality was considered when the lower limit of the 95% confidence interval (CI) for the ROR was >  1 and there were at least three records [12–14]. The level of statistical significance was established at P <  0.05. We also used mean, median, and interquartile ranges to describe the time to onset [15]. The onset time was determined by subtracting the treatment initiation date from the event onset date. The ratio imbalance measure calculation and formula (ROR) are expressed as follows:

**Table:** 

Category	Target adverse events	All other adverse events
Oseltamivir	A	b
Non-oseltamivir	C	d

Formula: ROR =  (a/b)/(c/d), 95% CI =  e^ln(ROR) ±  1.96(1/a +  1/b + 1/c +  1/d)∘0.5^.

ROR: reporting odds ratio, CI: confidence interval.

## Results

### General characteristics

**Fig 1:**
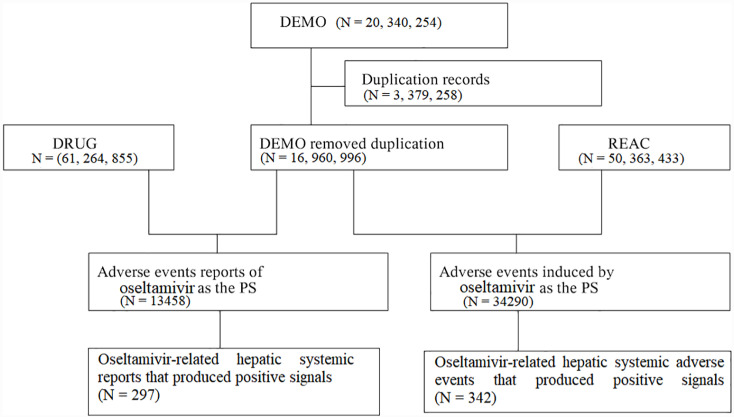
Flowchart of the study population. DEMO: Patient Demographics and Management, DRUG: Drug Details, REAC: Record of adverse events.

A total of 20,340,254 reports of adverse events were recorded by the FAERS during the study period (Fig 1). After deleting duplicates, 16,960,996 reports were included, with 13,458 reports indicating oseltamivir as the first suspicion for a total of 34,290 cases. Ultimately, 297 reports (342 instances) described hepatotoxic oseltamivir-related adverse events with a positive signal. Table 1 shows that 294 (98.99%) patients had a severe disease, 148 (49.83%) required hospitalization, 33 (11.11%) had a life-threatening disease, and 32 (10.77%) had a fatal disease. Health professionals published over 90% of the reports. Slightly more cases were reported among female individuals than among male individuals. The mean age was 47 years; children aged <  18 years were the fewest among the patients, and >  50% of the patients were aged 18–65 years. The top three reporting countries were Japan, France, and the United States, accounting for >  60% of the reports.

**Table 1:** Essential features of adverse events in oseltamivir-induced hepatotoxicity.

Characteristic	Count (%)
Sex	
Female (%)	141 (47.47)
Male (%)	135 (45.45)
Not specified or missing (%)	21 (7.07)
Age (years)	
< 18 (%)	35 (11.78)
≥ 18, < 45 (%)	75 (25.25)
≥ 45, < 65 (%)	76 (25.59)
≥ 65 (%)	67 (22.56)
Unknown (%)	44 (14.81)
Mean (SD)	47.00 (24.44)
Year reported (%)	
2004–2008	91 (30.64)
2009–2013	93 (31.31)
2014–2018	47 (15.82)
2019–2023 (Q3)	66 (22.22)
Types of reporter	
Physician (%)	185 (62.29)
Pharmacist (%)	50 (16.84)
Other health-professional (%)	38 (12.79)
Consumer (%)	22 (7.41)
Unknown (%)	2 (0.67)
Reported country (top 5)	
Japan (%)	105 (35.35)
France (%)	49 (16.50)
United States of America (%)	36 (12.12)
Not specified (%)	34 (11.45)
Spain (%)	17 (5.72)
Outcome	
Serious (%)	294 (98.99)
Non-serious (%)	3 (1.01)
Life-threatening	33 (11.11)
Hospitalization-initial or prolonged	148 (49.83)
Disability	6 (2.02)
Death	32 (10.77)
Required intervention to prevent permanent Impairment	1 (0.34)
Other	133 (44.78)

SD: standard deviation.

### Signal detection

**Fig 2:**
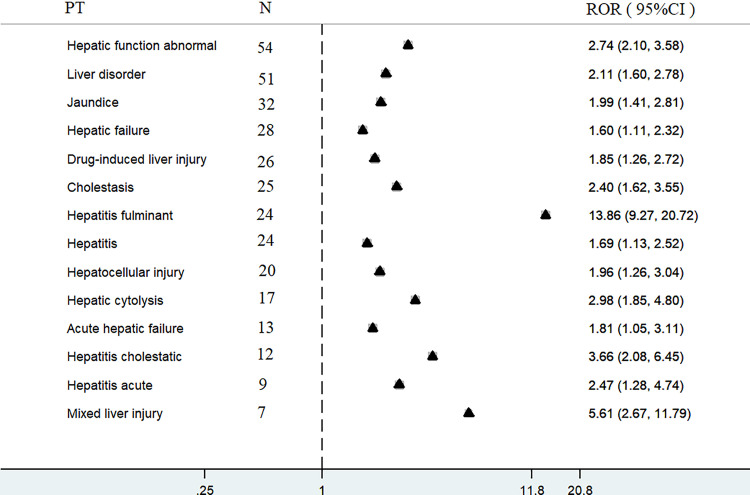
Forest plots of disproportionality of oseltamivir and hepatotoxicity. ROR: reporting odds ratio, CI: confidence interval.

Fig 2 shows the adverse events of oseltamivir-induced hepatotoxicity, with 14 adverse events exhibiting positive signals. The top five most reported cases were hepatic function abnormality (n =  54), liver disorders (n =  51), jaundice (n =  32), hepatic failure (n =  28), and drug-induced liver injuries (n =  26). The top five reported intensities, in descending order, were fulminant hepatitis, mixed liver injury, cholestatic hepatitis, abnormal hepatic function, and hepatic cytolysis. Their RORs were 13.86 (95% CI: 9.27–20.72), 5.61 (95% CI: 2.67–11.79), 3.66 (95% CI: 2.08–6.45), 2.98 (95% CI: 1.85–4.80), and 2.74 (95% CI: 2.10–3.58), respectively.

Sex differences in the 14 hepatotoxic adverse events are presented in Table 2. Hepatic function abnormality and drug-induced hepatic injury were significantly different between the sexes (P <  0.05). The P-values for cholestasis (P =  0.06) and fulminant hepatitis (P =  0.05) were close to but did not quite reach the level of significance, whereas the others were not significantly different between the sexes. The ROR decreased to 0.5 (95% CI: 0.29–0.86) for female individuals compared with that for male individuals, indicating a higher likelihood of abnormal hepatic function in male individuals. Conversely, female individuals were more likely to develop drug-induced liver injury than male individuals were, as the ROR increased to 4.19 (95% CI: 1.24–14.24) for female individuals compared with that for male individuals.

**Table 2:** Sex differences in oseltamivir-related hepatotoxicity adverse events (female vs. male).

Preferred term	Oseltamivir-related adverse events (Female)	All other drug-related adverse events (Female)	Oseltamivir-related adverse events (Male)	All other drug-related adverse events (Male)	ROR	95% CI	P
Hepatic function abnormality	22	17828	31	12439	0.50	0.29–0.86	0.01
Liver disorder	27	17823	22	12448	0.86	0.49–1.51	0.59
Jaundice	16	17834	13	12457	0.86	0.41–1.79	0.69
Cholestasis	10	17840	15	12455	0.47	0.21–1.04	0.06
Hepatic failure	12	17838	13	12457	0.64	0.29–1.41	0.27
Hepatitis fulminant	9	17841	14	12456	0.45	0.19–1.04	0.05
Hepatitis	14	17836	7	12463	1.40	0.56–3.46	0.47
Drug-induced liver injury	18	17832	3	12467	4.19	1.24–14.24	0.01
Hepatocellular injury	12	17838	7	12463	1.20	0.47–3.04	0.70
Hepatic cytolysis	10	17840	7	12463	1.00	0.38–2.62	1.00
Acute hepatic failure	5	17845	6	12464	0.58	0.18–1.91	0.55
Hepatitis cholestatic	4	17846	6	12464	0.47	0.13–1.65	0.37
Mixed liver injury	4	17846	3	12467	0.93	0.21–4.16	1.00
Hepatitis acute	2	17848	5	12465	0.28	0.05–1.44	0.21

CI, confidence interval.

**Fig 3:**
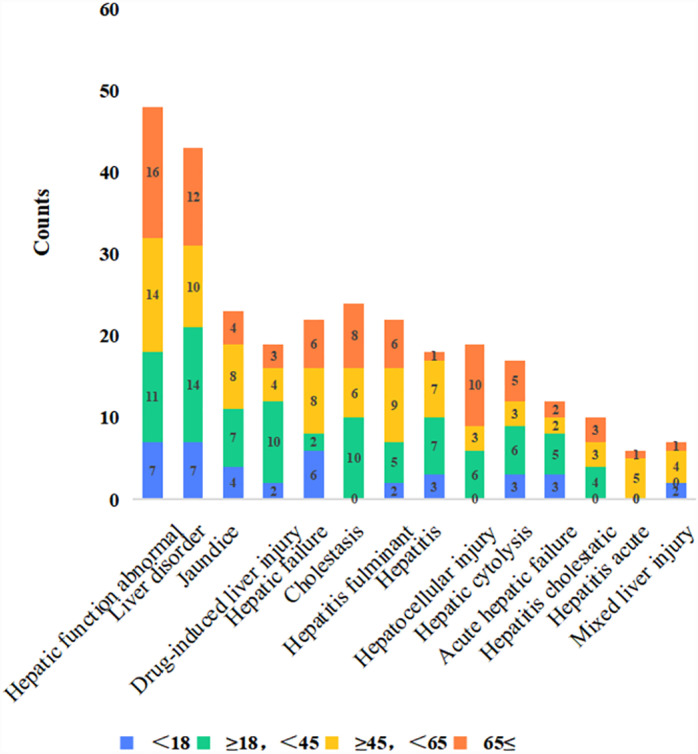
Distribution of adverse events related to oseltamivir-induced hepatotoxicity in different age groups.

Fig 3 presents the age differences among the 14 hepatotoxicity-related adverse events. The proportion of reported liver function abnormality increased with age. The most commonly reported liver injuries in patients aged 18–45 years were drug-induced liver injury, cholestasis, hepatic cytolysis, acute hepatic failure, and cholestatic hepatitis. Acute hepatitis and mixed liver injuries have not been reported. Jaundice, hepatic failure, fulminant hepatitis, and acute hepatitis were most frequently reported in patients aged 45–65 years. Cholestasis, hepatocellular injury, cholestatic hepatitis, and acute hepatitis were not observed in patients aged <  18 years. Liver function abnormality and liver disorder were most frequently reported in children aged <  18 years, whereas cholestasis, hepatocellular injury, cholestatic hepatitis, and acute hepatitis were not observed.

### Time of onset of events

**Fig 4:**
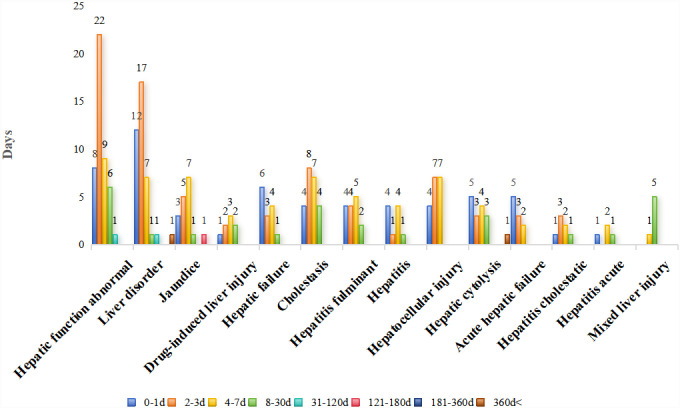
Distribution of oseltamivir-induced hepatotoxicity-related adverse events across various periods of occurrence.

**Fig 5:**
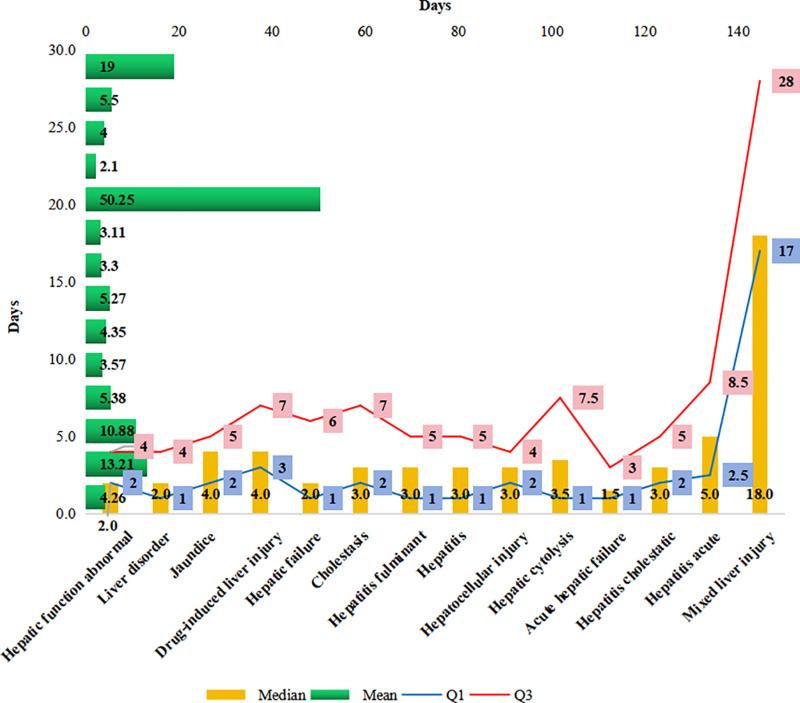
Time to onset of adverse events related to oseltamivir-induced hepatotoxicity (mean, median, and interquartile range).

Fig 4 illustrates the distribution of the number of reports regarding the 14 hepatotoxic adverse events at different periods of occurrence. Hepatic failure, hepatic cytolysis, and acute hepatic failure had the largest number of reports, with an onset time of 1 day; however, hepatocytolysis was also reported after 1 year. Abnormal hepatic function, liver disorders, cholestasis, and cholestatic hepatitis had the largest percentage of time of onset at 2–3 days, and the onset of liver disorders may also occur after 1 year. Jaundice, drug-induced liver injury, and fulminant hepatitis typically manifested within 4–7 days, with mixed liver injury specifically appearing after > 1 week, typically between 8 and 30 days. Fig 5 presents the mean, median, and interquartile range of the time to the occurrence of the 14 hepatotoxic adverse events. Acute liver failure had the shortest mean time to occurrence (2.10 days) and hepatocytolysis had the longest mean time to occurrence (50.25 days). The median time to acute liver failure was the shortest (1.5 days), and the median time to mixed liver injury was the longest (18 days). Excluding the median time to mixed liver injury, the median time to hepatotoxic adverse events was <  5 days.

## Discussion

Influenza has a significant impact on society, leading to high rates of absenteeism, increased healthcare burden, and elevated mortality among older adults and other vulnerable populations [16]. Therefore, ensuring active, safe, and effective prevention and treatment of influenza is a crucial medical priority. Although oseltamivir has been widely used for over two decades to prevent and treat influenza, with mostly mild side effects, recent case reports have highlighted the importance of monitoring adverse events, such as liver toxicity. The reported cases of liver toxicity related to oseltamivir are similar. However, comprehensive data on this issue are lacking. Through an analysis of global reports on hepatotoxicity, it was observed that oseltamivir may be associated with an increased risk of liver toxicity, emphasizing the need for careful management of oseltamivir use.

Hepatic impairment is a delayed adverse effect of oseltamivir, which may be related to the inhibition of host endogenous neuraminidase activity by oseltamivir carboxylate [8,11]. Over 2.2% (297/13458) of all adverse events reported for oseltamivir in the FAERS database were associated with hepatotoxicity. The use of oseltamivir was associated with a higher risk of 14 hepatotoxicity-related adverse events compared with the use of all other drugs listed in the FAERS database. The ROR for fulminant hepatitis was the highest. All were newly identified hepatotoxicity-related adverse events, except for hepatitis, hepatic function abnormality, and drug-induced liver injury, which had the highest number of reported cases, followed by liver disorders, jaundice, hepatic failure, and drug-induced liver injury.

Importantly, our study also found that the prevalence of liver function abnormality in female individuals was half that in male individuals. In contrast, the prevalence of drug-induced liver injury was 4.19-fold higher in female individuals than in male individuals. Except hepatic function abnormality and drug-induced liver injury, the prevalence of other adverse events did not differ by sex. Liver function abnormality refers to abnormalities in the metabolic, excretory, and synthetic functions of the liver, usually in the form of elevated levels of liver enzymes or other related indicators. Liver injury, on the other hand, refers to damage to the liver tissue itself. Liver function abnormality may be an early manifestation of liver injury; however, it does not necessarily lead to obvious damage to liver tissue. Drug-induced liver injury (DILI) is an impairment of liver function caused by exposure to a drug, excluding other factors. A proposed definition for DILI is established when one of the following thresholds is met [17]: (1) ALT levels reach five times the upper limit of normal (ULN); (2) ALP levels reach twice the ULN, particularly in conjunction with an elevation in gamma-glutamyl transferase or after excluding primary bone pathology in cases exhibiting isolated ALP elevation; (3) ALT levels are three times the ULN, in conjunction with a TBIL level exceeding twice the ULN.

None of the hepatotoxic adverse events occurred beyond 5 days from onset, except mixed liver injury, which typically occurred over 2 weeks later. Notably, liver disorders and hepatic cytolysis can manifest from the first day to up to a year later, showing a varied onset pattern, with no further adverse events reported beyond the 1-year mark. Healthcare providers are therefore advised to be vigilant for oseltamivir-induced hepatotoxicity when prescribing the drug, especially during the first week of empirical use of oseltamivir.

Exposure to various chemicals and biological agents can lead to liver disease. Some animal studies [18] have demonstrated that treating mice with oseltamivir significantly increases the expression levels of tumor growth factor-β1, interleukin-1β, matrix metalloproteinase-12, and collagen mRNA in the liver, resulting in hepatic side effects, heightened inflammation, and fibrosis. Furthermore, a clinical study [19] revealed that oseltamivir can cause liver function abnormality in humans, as indicated by elevated ALT and AST levels, with an occurrence rate of 0.1% (1 out of 771 patients). Although drug-induced liver injury is uncommon and approximately affects 14–19 out of 100,000 people annually [9], it remains a significant contributor to liver diseases. Furthermore, there is a documented case of oseltamivir-induced liver injury in a child in which prompt intervention led to the restoration of normal liver function [9], indicating that the liver-related effects of oseltamivir can be reversed in specific circumstances. Healthcare professionals must consider the potential risks of liver injury associated with antiviral therapy when evaluating liver damage during influenza, despite the rare reports of oseltamivir-induced liver injury in both children and adults. During influenza outbreaks, liver complications may arise along with respiratory issues, highlighting the importance of physicians in identifying pulmonary and extrapulmonary complications, including influenza-related liver complications and potential antiviral-induced liver injuries [8]. Early detection of oseltamivir-induced liver toxicity and prompt discontinuation of the drug are critical steps in managing such cases. Treatment with hepatoprotective agents, such as glycyrrhizin, reduced glutathione and glucocorticoids, is based on patient’s physical condition. Additionally, the precise mechanism by which oseltamivir causes liver injury remains unknown.

Oseltamivir is a prodrug of oseltamivir carboxylic acid, whose conversion is primarily performed by carboxylesterase 1 (CES1) in the liver [3,20]. The active metabolites are excreted mainly through urine. No interaction exists between the prodrug and its metabolite, glucuronosyltransferase, or cytochrome P450 mixed-function oxidase [21]. Although CES1 is expressed at various sites throughout the body, its highest expression is observed in the liver [22]. *In vitro* studies have shown the rapid metabolism of oseltamivir by human hepatic microsomes, with intestinal, plasma, and brain microsomes lacking hydrolytic activity [22]. Mastroianni et al [11] postulated a correlation between putative CES1 gene variants and possible oseltamivir-induced hepatotoxicity.

Regarding drug efficacy, no dose adjustment is required for oseltamivir in patients with mild-to-moderate hepatic impairment because of unchanged in vivo carboxylic acid oseltamivir exposure [23–25]. The adverse effects of oseltamivir may be related to its drug concentration [26]. It has been stated [20] that hepatic CES1 protein expression is 17.3% higher in female individuals than in male individuals (P =  0.039), indicating that oseltamivir activation is more pronounced in the livers of female individuals than in those of male individuals, potentially explaining the lower prevalence of hepatic function abnormality in female individuals than in male individuals. Furthermore, functional carboxylesterase 1 variants (e.g., G143E) influence oseltamivir activation in the 143G/E genotype. The rate of oseltamivir hydrolysis in livers with genotype 143G/E was approximately 40% that in livers with genotype 143G/G (P =  0.005). The CES1 variant G143E (rs71647871) is a nonsynonymous mutation caused by substituting a G residue for an A residue in exon 4, resulting in a change from glycine to glutamate at coding position 143. In Black, Hispanic, White, and Asian populations, the minor allele frequencies of G143E are 4.3%, 2.0%, 3.7%, and 0%, respectively [20]. The degree of liver damage, which affects CES1 protein expression, such as liver stiffness, alters the absorption and disposition of drugs, thereby affecting drug concentrations, particularly for drugs primarily metabolized by the liver [21]. Cirrhosis decreases CES1 expression, leading to significant exposure to oseltamivir and increased risk of toxicity [21]. Therefore, clinicians should be aware of the need to perform liver function tests and monitor for signs and symptoms of liver disease in cases of severe liver damage or when other hepatotoxic drugs must be co-administered.

In addition to oseltamivir, zanamivir and peramivir are also considered essential medications for the treatment of influenza [27]. The label for zanamivir indicates that it is not metabolized in the liver and therefore no dose adjustment is required [28]. Zanamivir exhibits negligible hepatic metabolism and does not influence cytochrome P450 activity. Additionally, it is not hepatotoxic, owing to its generally short duration of therapy, brief exposure period during inhalation administration, and minimal hepatic metabolism [29]. Similarly, peramivir exhibits minimal metabolism within the human body, with approximately 90% being excreted unchanged in the urine. It is noteworthy that peramivir does not serve as a substrate for the cytochrome P450 enzyme system nor does it substantially interact with other pharmacological agents. Furthermore, given that peramivir is typically administered via a single intravenous infusion, the risk of hepatotoxicity is negligible due to the brief exposure duration and limited hepatic metabolism [30]. For patients with influenza who also exhibit severe liver damage, the lack of clinical studies on the use of oseltamivir in this specific population suggests that peramivir and zanamivir may represent more suitable alternatives.

This study has some limitations. This was a retrospective study of events reported in the FAERS; therefore, the basic hepatic characteristics of patients taking oseltamivir were unknown. In addition, the actual hepatic toxicity of oseltamivir could not be determined because not all events were reported in the FAERS database. Therefore, the hypotheses proposed in this study need to be validated in future prospective studies.

## Conclusions

The FAERS pharmacovigilance analysis revealed an elevated risk of 14 hepatotoxicity-related adverse events associated with oseltamivir. Notably, fulminant hepatitis had the highest ROR, and the largest number of cases of abnormal hepatic function was documented as associated with the use of oseltamivir compared with the other drugs in the FAERS. The incidence of hepatic function abnormality in female individuals was half as that in male individuals, whereas drug-induced liver injury was 4.19 times more prevalent in female individuals. Taken together, it is not recommended to discontinue oseltamivir if liver damage occurs while taking it. Monitoring of liver function is recommended when oseltamivir is used concomitantly with other potentially hepatotoxic drugs.
